# Immunosuppressive Activity of Size-Controlled PEG-PLGA Nanoparticles Containing Encapsulated Cyclosporine A

**DOI:** 10.1155/2012/896141

**Published:** 2012-03-29

**Authors:** Li Tang, Jamil Azzi, Mincheol Kwon, Marwan Mounayar, Rong Tong, Qian Yin, Robert Moore, Nikolaos Skartsis, Timothy M. Fan, Reza Abdi, Jianjun Cheng

**Affiliations:** ^1^Department of Materials Science and Engineering, University of Illinois at Urbana-Champaign, Urbana, IL 61801, USA; ^2^Transplantation Research Center, Renal Division, Brigham and Women's Hospital; Children's Hospital Boston, Harvard Medical School, Boston, MA 02139, USA; ^3^Department of Veterinary Clinical Medicine, University of Illinois at Urbana-Champaign, Urbana, IL 61801, USA

## Abstract

We encapsulated cyclosporine A (CsA) in poly(ethylene glycol)-*b*-poly(d,l-lactide-co-glycolide) (PEG-PLGA) nanoparticles (NPs) by nanoprecipitation of CsA and PEG-PLGA. The resulting CsA/PEG-PLGA-NPs were <100 nm in diameter with a narrow particle size distribution. The NP size could be controlled by tuning the polymer concentration, solvent, or water/solvent ratio during formulation. The PEGylated NPs maintained non-aggregated in salt solution. Solid NPs lyoprotected with bovine serum albumin were prepared for the convenience of storage and transportation. The release kinetics of CsA (55.6% released on Day 1) showed potential for maintaining therapeutic CsA concentrations *in vivo*. In T-cell assays, both free CsA and CsA/PEG-PLGA-NPs suppressed T-cell proliferation and production of inflammatory cytokines dose dependently. In a mixed lymphocyte reaction assay, the IC_50_ values for free CsA and CsA/PEG-PLGA-NPs were found to be 30 and 35 ng/mL, respectively. This nanoparticulate CsA delivery technology constitutes a strong basis for future targeted delivery of immunosuppressive drugs with improved efficiency and potentially reduced toxicity.

## 1. Introduction

The efficacy of immunosuppressive drugs has markedly improved over the last 2 decades, and as a result, the 1-year graft survival rate now exceeds 80% for most solid organ transplant. However, only modest improvements of long-term graft survival rates have been achieved, owing mainly to the systemic toxicity of immunosuppressants. When calcineurin inhibitors were introduced in mid-1980s, they revolutionized the field of transplantation by substantially reducing the rate of acute rejection [1, 2]. In particular, the discovery of cyclosporine A (CsA), a highly lipophilic neutral cyclic peptide, was a milestone in organ transplantation and the treatment of autoimmune diseases.

CsA effectively suppresses immune reactions dependency on T cells, which are the key effector cells involved in graft rejection and autoimmunity. It forms a complex with cyclophilin, a cytoplasmic receptor protein present in T lymphocytes, and this complex then binds to calcineurin and inhibits Ca^2+^-stimulated dephosphorylation of the cytoplasmic component of the nuclear factor of activated T cells. This factor regulates transcription of numerous genes involved in T-cell activation and proliferation, such as IL-2, IL-4, and CD40 ligand [14]. When these genes are not expressed, T-cell-dependent immune responses are dramatically inhibited. However, CsA exhibits low oral bioavailability owing to its poor biopharmaceutical properties, such as its high molecular weight (1202 Da), low solubility, low permeability, and high presystemic metabolism [3, 4]. Consequently, there is an urgent need for the design and development of a novel formulation of CsA with better bioavailability and fewer side effects.

Nanotechnology has been increasingly used in medicine [5]. For example, nanoparticulate formulations of drugs can extend their blood circulation and offer new methods for targeted drug delivery after intravenous administration. Several nanoparticulate CsA formulations, such as emulsions, liposomes, microspheres, and polymeric nanoparticles (NPs), have been developed to compensate for the poor biopharmaceutical properties of CsA and potentially reduce its side effects through specific targeting and controlled release of CsA [6, 7]. In particular, polymeric NPs based on polylactide (PLA) or poly(lactide-co-glycolide) (PLGA) have attracted much attention as delivery vehicles for CsA because of their excellent biocompatibility [8–11]. The nanoparticulate formulations developed to date have improved the overall bioavailability of CsA and protected the milieu which surrounds the disease site from the toxicity of CsA. Many of the published studies have focused on controlling the formulation for maintaining therapeutic blood levels of CsA or for achieving favorable biodistribution. However, if clinically relevant NP-encapsulated CsA are to be developed successfully, their immunosuppressive capability must be directly demonstrated and assessed.

In a recent report, we described a novel formulation of CsA-PLA covalently conjugated with NPs for targeted immunosuppression [12]. This new formulation suppresses T-cell proliferation both *in vitro* and *in vivo* and shows reduced organ toxicity. However, the release of CsA from the polymeric NPs is relatively slow (only ~20% of the CsA is released over 2 weeks). Therefore, maintenance of CsA concentrations within the therapeutic window after intravenous administration is difficult with this formulation. In an attempt to increase the effective concentration of CsA delivered by the NPs, we encapsulated CsA in size-controlled poly(ethylene glycol)-*b*-poly(d,l-lactide-co-glycolide) (PEG-PLGA) NPs instead of using the covalent conjugation strategy. Owing to the absence of covalent bonds between CsA and the polymer matrix, we expected this encapsulation strategy to permit more effective release of CsA to achieve the desired CsA concentration. In the meantime, the controlled release of CsA can reduce the *C*_max⁡_ of CsA while keeping the same AUC [13].

To demonstrate and assess the immunosuppressive activity of CsA-containing PEG-PLGA NPs, we designed several *in vitro* assays to directly evaluate the capacity of this formulation to suppress the activation and proliferation of T cells *in vitro*. We also investigated the control of NP sizes of the CsA-containing PEG-PLGA NPs. Because the immunosuppressive activity of CsA is related to its selective action against T lymphocytes, which circulate mainly in the lymphatic system, effective targeting of the lymphatic system is clearly important for the delivery of CsA. Some recent reports have emphasized that smaller NPs more effectively target the lymphatic system [15, 16]. Moreover, smaller NPs may also minimize the burst release of CsA from NPs [11]. However, size control of NP delivery systems for CsA has been rarely reported. Recently, we used PEG-PLGA to formulate size-controlled NPs for delivery of anticancer drugs [17]. Owing to the facile formulation procedure and excellent control over NP size, we applied similar techniques in this study to obtain size-controlled PEG-PLGA NPs encapsulating CsA. These PEG-PLGA NPs encapsulating CsA were stable in biological media with negligible aggregation. To ensure the potential clinical translation, NPs in solid form with unchanged properties is preferred. In this study, we tested and demonstrated that solid form of CsA/PEG-PLGA NP can be prepared by using bovine serum albumin (BSA) as a lyoprotectant.

## 2. Materials and Methods

### 2.1. General

Cyclosporine A (LC Laboratories, Woburn, MA, USA) was used as received. All other chemicals were obtained from Sigma-Aldrich (St. Louis, MO) unless otherwise noted. Poly(ethylene glycol)-*b*-poly(d,l-lactide-co-glycolide) was purchased from Laysan Bio (Arab, AL, USA). All anhydrous solvents were passed through alumina columns and kept anhydrous over molecular sieves. HPLC analysis was performed on a System Gold system (Beckman Coulter, Fullerton, CA, USA) equipped with a 126P solvent module (Beckman), a System Gold 128 UV detector (Beckman), and an analytical column (Jupiter, 250 × 4.6 mm, 5 *μ*m, Phenomenex, Torrence, CA, USA). The UV wavelength used for detection was 207 nm. The size and size dispersity of nanoparticles were characterized on Hitachi S4800 high-resolution scanning electron microscope (SEM). NP sizes and dispersities were also monitored in real time with a Zeta PALS dynamic light-scattering (DLS) detector (15 mW laser, incident beam = 676 nm, Brookhaven Instruments, Holtsville, NY, USA). NPs were lyophilized on a benchtop lyophilizer (Freezone 2.5, Fisher Scientific, Pittsburgh, PA, USA).

### 2.2. Preparation of CsA-Containing PEG-PLGA NPs (CsA/PEG-PLGA-NPs)

The average molecular weight and polydispersity of PEG-PLGA were 16,400 g/mol and 1.19, respectively, as determined by size-exclusion chromatography coupled to an Optilab rEX refractive index detector and a DAWN HELEOS 18-angle laser light scattering detector (MALLS, Optilab rEX refractive index detector). A nanoprecipitation method was used to formulate CsA-containing PEG-PLGA NPs. Briefly, PEG-PLGA (157.3 mg) was added to an acetone solution of CsA (4.9 mg, 0.004 mmol) to make the concentration of PEG-PLGA 10 mg/mL. This solution (1.0 mL) was added dropwise into 20 mL nanopure water under vigorous stirring to formulate CsA-encapsulated PEG-PLGA NPs. The NP suspension was stirred uncovered for 6 h at room temperature in a chemical hood in order to completely evaporate the organic solvent. An aliquot of the NP suspension was centrifuged (10 min, 10 k rpm) and the supernatant was analyzed by a reverse-phase HPLC to quantify the unencapsulated CsA in order to determine the incorporation efficiency and loading of CsA in PEG-PLGA NPs. The NPs were purified and collected by ultrafiltration (15 min, 3000 rpm, Amicon Ultra, Ultracel membrane with 100,000 NMWL, Millipore, Billerica, MA, USA) and lyophilized. The size and polydispersity of the resulting NPs were determined by DLS and SEM.

### 2.3. Size Control of CsA/PEG-PLGA-NPs

Parameters controlling formulation of the NPs were systematically varied in this study. The nanoprecipitation method was employed for the formation of CsA-encapsulated PEG-PLGA NPs. Generally, the starting formulation was as follows: PEG-PLGA (10 mg/mL) and CsA (0.31 mg/mL) were dissolved in *N,N*-dimethylformamide (DMF). The mixture was added dropwise to a 20-fold volume of vigorously stirred nanopure water. The NPs were produced with the nanoprecipitation method in a water-miscible solvent, such as: DMF, acetone, and tetrahydrofuran (THF). The effects of the various solvents were assayed on the overall size of the NPs. For each solvent, a range of polymer concentrations in the organic phase from 1 mg/mL to 10 mg/mL was used for the formation of the NPs in a 20-fold volume of water. Then, polymer concentration was fixed at 10 mg/mL; the effect of volume ratio of water/organic solvent (5/1, 10/1, 20/1, 40/1) was studied.

### 2.4. Stability of CsA/PEG-PLGA-NPs in Salt Solution

The postformulation stability of NPs in PBS (1×) was studied for both poly(ethyleneglycol)ated (PEGylated) and non-PEGylated NPs. PEGylated NPs were formed by precipitating 100 *μ*L of a DMF solution of PEG-PLGA (10 mg/mL) and CsA (0.31 mg/mL) to DI water (2 mL) under vigorous stirring. For non-PEGylated NPs, 100 *μ*L of a DMF solution of PLGA (10 mg/mL) and CsA (0.31 mg/mL) was used instead. Afterwards, 10× PBS solution was added to bring the whole mixture to 1× PBS. The NP sizes in PBS were measured by DLS and followed over 30 min.

### 2.5. Lyophilization of CsA/PEG-PLGA-NPs in the Presence of BSA as a Lyoprotectant

Bovine serum albumin (BSA) as a lyoprotectant was added to the concentrated NP suspension at a BSA/NP mass ratio of 10/1. The solution was then lyophilized to dry powder, which was stored at −20°C prior to use. The NPs were reconstituted with water and stirred for 5 min. The sizes of the resulting NPs were analyzed by DLS.

### 2.6. Determination of the Release Kinetics of CsA/PEG-PLGA-NPs

The solution of CsA/PEG-PLGA-NPs (0.43 mg/mL) was incubated in 1× PBS at 37°C. At scheduled time intervals (*t* = 0, 1, 2, 3, and 4 days), the incubated NP solutions were taken out and lyophilized overnight to dry. Ether was added to extract all the released CsA. After the solvent was evaporated, 500 *μ*L acetonitrile (ACN) was added. This solution was analyzed by HPLC to quantify the released CsA. The release kinetics of CsA was determined by comparing the released CsA versus the total CsA, which was calculated by the loading information.

### 2.7. Mixed Lymphocyte Reaction and CD3/CD28 T-Cell Stimulation Assays

The mixed lymphocyte reaction (MLR) assay was performed as described previously [18]. Briefly, in 96-well U-bottom plates, 5 × 10^5^ each of responder and irradiated stimulator splenocytes were cultured in triplicates in the presence of increasing dosages of free CsA and CsA/PEG-PLGA-NPs (free CsA and CsA/PEG-PLGA-NPs were added at the same equivalent dosage of CsA) for 48 hours. Another group of PEG-PLGA-NPs without CsA was added at the same total mass concentration of NP as negative control. Cultures were pulsed with 1 *μ*Ci of triturated thymidine and the incorporation efficiencies were determined. Data are expressed as the mean cpm of the [H] thymidine uptake by triplicate cultures plus the standard deviation. For the CD3/CD28 stimulation assay, 100 *μ*L of anti-CD3 antibody in PBS (1 ug/mL, eBioscience, San Diego, CA, USA) was added to each well of a 96-well flat bottom plate, incubated at 37°C for 4 hours, and then washed twice with PBS. Soluble anti-CD28 antibody (1 *μ*g/mL, eBioscience) was added to each well in the presence of 5 × 10^5^ responder splenocytes and an increasing concentration of free CsA, CsA/PEG-PLGA-NPs, and PEG-PLGA-NPs. Cultures were pulsed with triturated thymidine as described above.

### 2.8. ELISpot Assay

The enzyme-linked immunosorbent spot (ELISpot) was performed as previously described by us [19]. Briefly, ELISpot plates were coated with capture antibodies against interferon (IFN-*γ*). A total of 5 × 10^5^ splenocytes were cultured in triplicate in the presence of the same number of irradiated allogeneic splenocytes. Irradiated syngeneic splenocytes and Con A were used as negative and positive controls, respectively. Different concentrations of free CsA, CsA/PEG-PLGA-NPs, and PEG-PLGA-NPs were added to the wells. After 48 hours, the resulting spots were counted on a computer-assisted ELISAspot image analyzer (Cellular Technology).

## 3. Results

### 3.1. Preparation of CsA/PEG-PLGA-NPs

**Figure 1:**
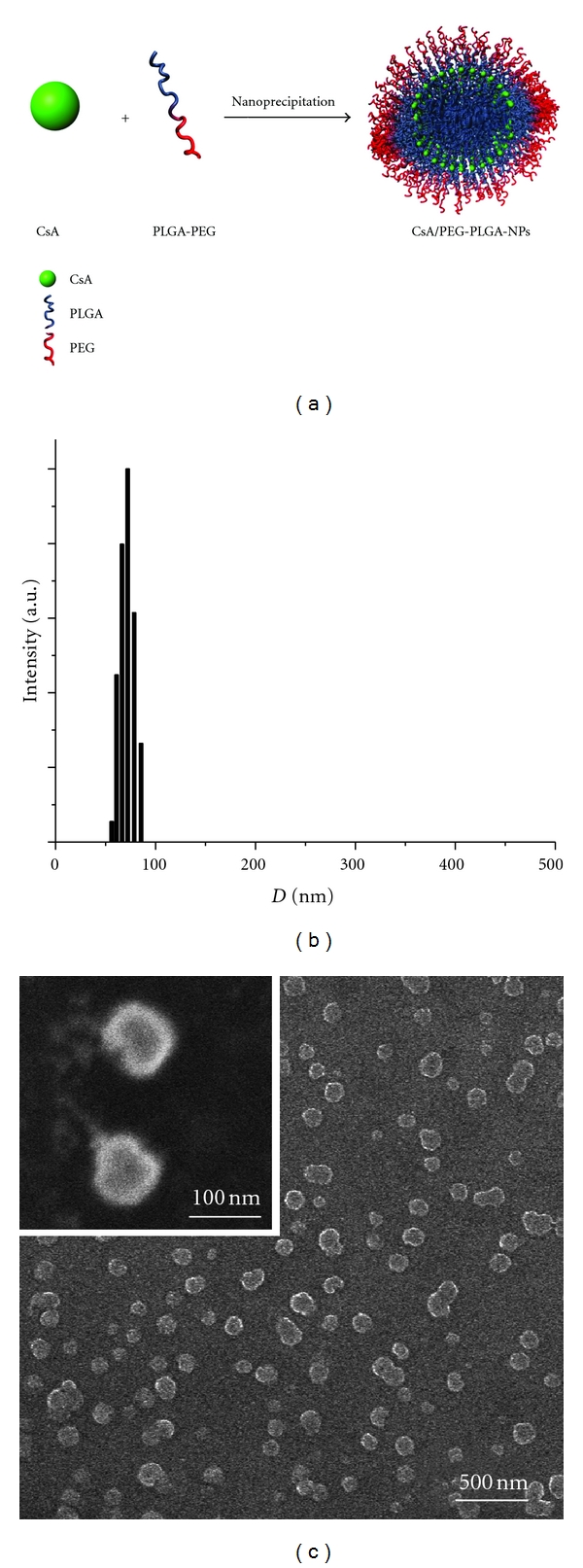
(a) Preparation of CsA/PEG-PLGA-NPs through nanoprecipitation. DLS (b) and SEM (c) characterization of the sizes of the CsA/PEG-PLGA-NPs.

When CsA was mixed with the amphiphilic block polymer PEG-PLGA in an organic solvent, and the mixture was added dropwise to a nonsolvent (e.g., water), the hydrophobic domain of PEG-PLGA (PLGA) formed a hydrophobic core with CsA being encapsulated (Figure 1(a)). The hydrophilic domain, PEG, remained on the surface of the polymeric NPs and stabilized the NP size. The diameter of the prepared CsA/PEG-PLGA-NPs was 83.3 ± 0.8 nm with a monomodal particle size distribution and low polydispersity (Figure 1(b)). The narrow, monomodal particle size distribution was confirmed by SEM analysis (Figure 1(c)). Spherical sub-100-nm NPs could be seen clearly by SEM. The CsA loading was determined to be 3.3% (w/w) by HPLC. 

### 3.2. Control of the Size of the CsA/PEG-PLGA-NPs Prepared via Nanoprecipitation

**Figure 2:**
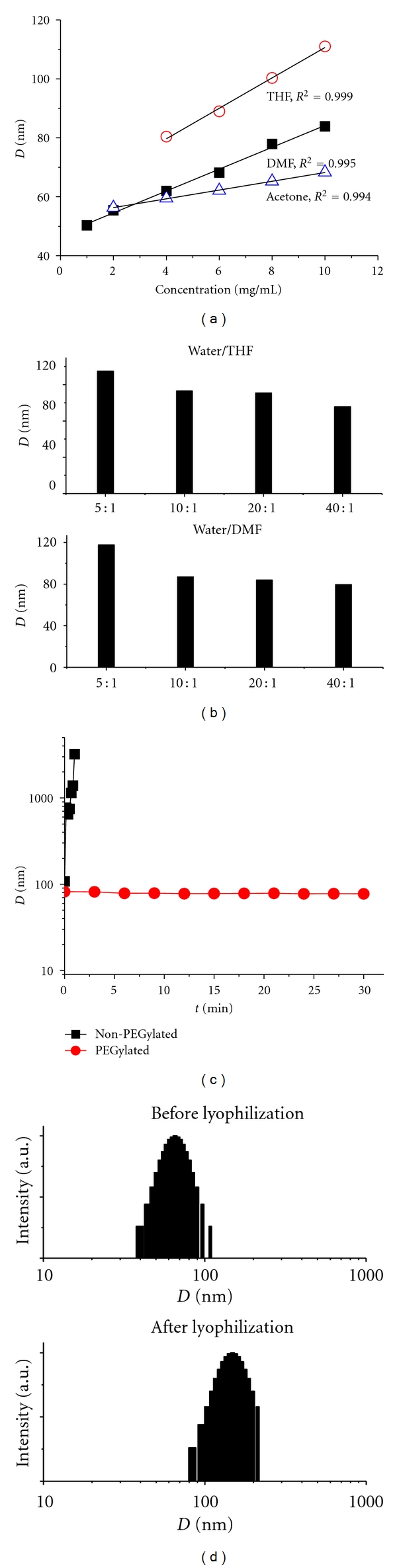
Controlled formulation of CsA/PEG-PLGA-NPs. Correlation of NP sizes versus polymer concentrations in different organic solvents at constant ratio of volume of solvent to water. NP sizes increased from 42.3 nm to 91.4 nm as the polymer concentration increased from 1 mg/mL to 10 mg/mL in DMF. Similar trends were observed in acetone and THF (a). Correlation of NP sizes versus ratio of volume of solvent to water in different organic solvents at constant polymer concentrations (10 mg/mL) (b). The PEGylated CsA/PEG-PLGA-NPs kept their original size and remained nonaggregated for an extended period of time. However, when the non-PEGylated NPs were tested in PBS (1×), they were unstable and formed large aggregates rapidly (the particle size increased from 108.1 nm to 3223.1 nm within 1 min) (c). The size of NPs increased slightly but maintained a single distribution after lyophilization with BSA as the lyoprotectant (d).

When the PEG-PLGA concentration was varied during NP preparation at a fixed water/solvent ratio of 20/1 (Figure 2(a)), NP size increased linearly with increasing polymer concentration (*R* ^2^ = 0.995 in DMF). Specifically, the NP size increased from 50.3 to 83.9 nm in DMF as the polymer concentration was increased 10 folds (from 1 to 10 mg/mL). Similar trends were observed in acetone (*R* ^2^ = 0.994) and in THF (*R* ^2^ = 0.999). The NPs formulated with THF were larger than those formulated with either DMF or acetone at the same polymer concentration. 

The water miscibility of the organic solvent also affected the size of the NPs [17, 20, 21]. Increasing the water miscibility led to a decrease in the mean NP size when all other formulation parameters were held constant. NPs prepared in THF, the least water-miscible of the tested solvents, had the largest particle size (Figure 2(a)). Because acetone, unlike DMF, can be easily removed by evaporation and the NPs prepared with acetone were comparable in size to the NPs prepared with DMF, we used acetone as the solvent for CsA and PEG-PLGA in the nanoprecipitation of CsA/PEG-PLGA-NPs.

At a fixed polymer concentration of 10 mg/mL (Figure 2(b)), the NP size decreased with increasing water/solvent ratio: for example, NP size decreased from 117.7 to 79.4 nm when the water/DMF ratio was increased from 5/1 to 40/1. In comparison, the NP sizes were 134.9 and 95.6 nm when the water/THF ratios were 5/1 and 40/1, respectively. For both solvents, when the water/solvent ratio was decreased from 10/1 to 5/1, a large increase in NP size (~20–30 nm) was observed.

### 3.3. Stability of CsA/PEG-PLGA-NPs in Salt Solution

Polymeric NPs tend to aggregate under biological conditions. To achieve prolonged systemic circulation and disease targeting, NPs should remain nonaggregated under physiological conditions. One method for preventing aggregation is to modify NP surfaces with PEG (PEGylation) [17, 22, 23]. To mimic the behavior of the NPs under physiological conditions, we evaluated NP stability in PBS. After precipitating a DMF solution of CsA and PEG-PLGA in vigorously stirred DI water and subsequently tuning the solution to 1× PBS, the NPs remained nonaggregated for at least 30 min, evidenced by the DLS analyses (Figure 2(c)). In contrast, non-PEGylated NPs (CsA-containing PLGA NPs) rapidly formed aggregates in PBS (Figure 2(c)); the particle size increased from 108 nm to over 3 *μ*m within 1 min. The enhanced stability of the CsA/PEG-PLGA-NPs in PBS suggests that they may show long circulation profile *in vivo*.

### 3.4. Control of NP Size during Postformulation Treatment

The stability of any biodegradable formulation upon storage is a concern for clinical use. Freeze drying NPs and then storing them in the solid state is a common approach for maintaining stability. We previously reported that albumin is an excellent lyoprotectant for the preparation of NPs in solid form, allowing lyophilized NPs to be readily dispersed in PBS (1×) without noticeable formation of aggregates [24]. In this study, the addition of a 10-fold excess mass of BSA to an aqueous suspension of CsA/PEG-PLGA-NPs resulted in recovery of NPs that were slightly larger than the original NPs (Figure 2(d)); these NPs which are in solid form could be readily dispersed in PBS (1×) without any aggregation. Without BSA as a lyoprotectant, the NPs formed aggregates that were several to tens of micrometers in size (data not shown) and could not be redispersed in PBS (1×). Thus, they were not useful upon reconstitution for *in vivo* systemic delivery.

### 3.5. Determination of CsA/PEG-PLGA-NPs Release Kinetics

**Figure 3:**
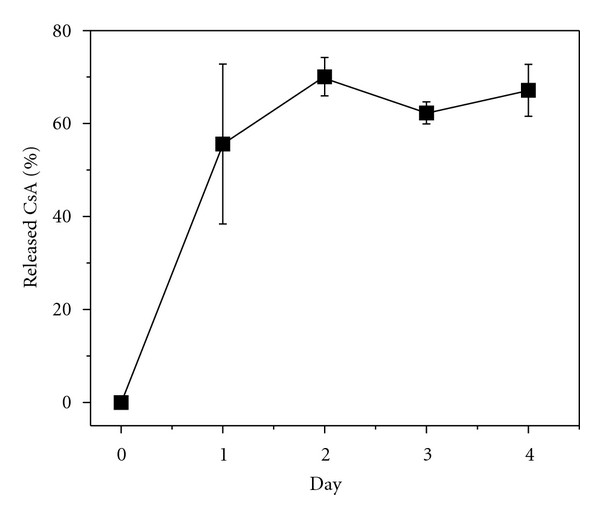
The release profile of CsA from CsA/PEG-PLGA-NPs in PBS (1×) at 37°C.

We recently reported that CsA release from CsA-PLA conjugated NPs (8.0 wt%) proceeds in a controlled but slow fashion, with 14.1% being released by Day 4 and 21.0% by Day 14 [12]. For this CsA-encapsulated PEG-PLGA NPs, CsA release was much faster compared to CsA release from the conjugated NPs because CsA was physically encapsulated inside the polymeric NPs without any covalent bonding. Up to 70% of CsA was released by Day 2 (Figure 3).

### 3.6. Suppression of T-Cell Proliferation by CsA/PEG-PLGA-NPs

**Figure 4:**
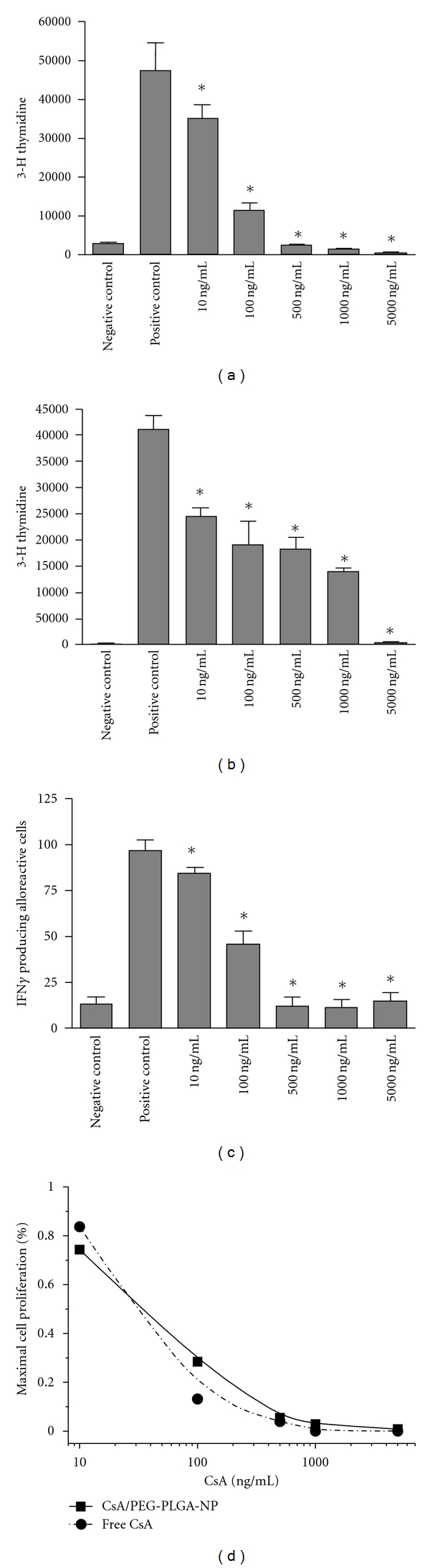
The CsA/PEG-PLGA-NPs suppress T-cell activation in MLR, CD3-CD28, and ELISpot assays. (a) CsA/PEG-PLGA-NPs showed dose-dependent inhibition of T-cell proliferation in an MLR assay starting at 10 ng/mL equivalent concentration of CsA. (b) CsA/PEG-PLGA-NPs suppressed T-cell proliferation in a CD3-CD28 stimulation assay in a dose-dependent manner starting at 10 ng/mL. PEG-PLGA-NPs was unable to suppress. Data are expressed as the mean cpm of the [H] thymidine uptake by triplicate cultures and represented by the *y* axis. The different concentrations used are represented by the *x* axis (*P < 0.05). Data are representative of two separate experiments. (c) The incidence of cells producing IFN-*γ* was first measured by ELISpot assay from cultured splenocytes of C57Bl/6 animals responding to BALB/c stimulating splenocytes *in vitro*. As compared to the positive control (untreated stimulated cells), CsA-NPs reduced the frequency of IFN-*γ*-producing cells in a concentration-dependent manner (*P < 0.05). Data are representative of two separate experiments (*P < 0.05). (d) IC_50_ calculation using the percentage of cell proliferation in a MLR assay, measured by thymidine uptake in response to increasing dose of free CsA and CsA equivalent of CsA/PEG-PLGA-NPs (*P < 0.05).

To compare the immunosuppressive abilities of CsA/PEG-PLGA-NPs and free CsA in an *in vitro* model relevant to transplantation, we added CsA/PEG-PLGA-NPs, free CsA, and PEG-PLGA-NPs at various concentrations to an MLR assay system. As compared to the positive control, free CsA dose dependently inhibited splenocyte proliferation in the MLR assay. The same pattern of suppression was observed for CsA/PEG-PLGA-NPs (Figures 4(a) and 4(d)). In contrast, equivalent concentrations of PEG-PLGA NPs did not suppress T-cell proliferation (data not shown).

We also evaluated the immunosuppressive effects of the NPs by means of a CD3-CD28 stimulation assay, which has traditionally been used to test the immunosuppressive effects of newly introduced drugs for the treatment of various immune-mediated diseases [25]. The results of the CD3-CD28 assay were similar to those of the MLR assay: CsA/PEG-PLGA-NPs dose dependently suppressed T-cell proliferation similar to free CsA (Figure 4(b)). The IC_50_ values for free CsA and CsA/PEG-PLGA-NPs, calculated from the suppression of T-cell proliferation in the MLR assay, were 30 and 35 ng/mL, respectively (Figure 4(d)). The higher IC_50_ of CsA/PEG-PLGA-NPs is consistent with the controlled release of CsA/PEG-PLGA-NPs. PEG-PLGA NPs had no effect on cytokine production (data not shown).

### 3.7. Suppression of Inflammatory Cytokine Production by CsA/PEG-PLGA-NPs

In addition to testing the effects of the NPs on T-cell proliferation, we also tested their effects on the pattern of IFN-*γ* production by activated T cells, which plays an important role in the pathogenesis of T-cell-mediated diseases [26–28]. The frequency of IFN-*γ*-producing cells was measured by means of an ELISpot assay with cultured splenocytes of C57Bl/6 mice in response to stimulation by irradiated BALB/c splenocytes *in vitro* in the presence of increasing doses of either free CsA or CsA/PEG-PLGA-NPs (Figure 4(c)). ELISpot is a sensitive, highly reproducible assay for measuring IFN-*γ* production, and is often used to examine alloreactive T-cell priming in the context of transplantation. All assays showed significant response to concanavalin A, indicating adequate viability of these cells. The number of spots in the wells with syngeneic splenocytes was used as the negative control. In all cases, the number of background spots was considered when analyzing the data. As compared to the positive control (untreated stimulated cells), both free CsA and CsA/PEG-PLGA-NPs comparably reduced the frequency of IFN-*γ*-producing cells in a concentration-dependent manner starting at 10 ng/mL (Figure 4(c); *P* < 0.05). No suppression was observed with PEG-PLGA-NPs.

## 4. Discussion

Nanoprecipitation is extensively used for the preparation of NPs with therapeutic agents embedded in the hydrophobic polymeric matrices. This method allows for rapid access to NPs in large quantities [17]. Typically, a mixture of a hydrophobic polymer and the drug is dissolved in a water-miscible organic solvent (e.g., DMF or acetone), and then the solution is added dropwise to a vigorously stirred water solution. Instantaneous diffusion of the organic solvent into the water results in the formation of polymeric NPs containing the drug. Optimization of the properties of the drug and polymer is important for efficient encapsulation of the drug. CsA, which is highly lipophilic, is suitable for incorporation into a hydrophobic polymer matrix to form CsA-containing polymeric NPs by means of nanoprecipitation. In this study, we used nanoprecipitation to prepare CsA-containing PEG-PLGA NPs and found that the hydrophobic polymer segment (i.e., PLGA) shielded the drug from the external environment, thus limiting its toxicity and allowing its coupling to targeted cellular therapy.

Particle size is one of the most important parameters of NPs and strongly influences their biodistribution, clearance kinetics, and *in vivo* efficacy [29–31]. The development of strategies for precisely controlling particle size is of interest for both basic research and clinical applications. Here, we were able to control the size of CsA/PEG-PLGA-NPs by tuning the polymer concentration, solvent, or water/solvent ratio during the formulation process. Previous studies by others [20, 32] and by us [17] have suggested that the miscibility of the organic solvent in water affects NP size in a given solvent/water system. Generally, the miscibility can be quantitatively expressed by comparing the solubility parameters (*δ*) of both the solvent and water [33]. As shown in Figure 2(a), the size of the CsA/PEG-PLGA-NPs and the water miscibility of the organic solvents used in this study were inversely correlated: an increase in the water miscibility (a decrease in the difference of solvents' solubility parameter Δ*δ*) led to a decrease in the mean size of the NPs when all the other formulation parameters were held constant. Smaller NPs were prepared in DMF or acetone than THF because they are more water miscible than THF and thus have more efficient solvent diffusion and polymer dispersion into water.

In a recent paper, we reported the CsA-release kinetics of CsA-PLA conjugated NPs [12]. Because CsA release from the conjugated NPs involves not only diffusion but also hydrolysis of the ester linker between CsA and the polymer, CsA release from the conjugated NPs is much slower than the encapsulated formulation. CsA is released from CsA-PLA NPs slowly and in a controlled fashion, with 14.1% being released by day 4 and 21.0% by day 14. In contrast, the CsA/PEG-PLGA-NPs released CsA much more quickly, mainly because there were no covalent bonds between CsA and the polymer matrix and the release kinetics were controlled solely by diffusion.

Finally, we tested the ability of the CsA/PEG-PLGA-NPs to suppress T-cell activation *in vitro*. We first evaluated the immunosuppressive effects of the NPs by means of a CD3-CD28 stimulation assay, which have traditionally been used to test the immunosuppressive effects of newly introduced drugs for the treatment of various immune-mediated diseases [25]. The released CsA inhibited T-cell proliferation in a dose-dependent manner, not only in a CD3/CD28 assay but also in an alloreactive MLR assay. IFN-*γ* production by alloreactive T cells was also suppressed by the CsA/PEG-PLGA-NPs in a dose-dependent manner, and the suppression was comparable to that observed for free CsA. Nanoparticles injected intravenously could accumulate in tumors via the enhanced permeability and retention (EPR) effect. Our CsA/PEG-PLGA NP could be potentially used to deliver cyclosporine to inflamed lymphoid tissues via the similar effect [34]. NPs preferentially accumulate at target sites of inflammation due in part to increased vascular permeability.

## 5. Conclusions

We formulated CsA-encapsulated PEG-PLGA NPs with controlled sizes and excellent stability in PBS. When BSA was used as the lyoprotectant, the CsA-encapsulated PEG-PLGA NPs could be prepared in the solid form without noticeable change of properties. CsA released from the CsA/PEG-PLGA-NPs suppressed the proliferation of T cells and the production of inflammatory cytokines *in vitro*. With the use of this controlled release technology in conjunction with precisely controlled particle size, this immunosuppressive nanomedicine showed well-maintained *in vitro* therapeutic efficacy. In the future work, we will evaluate the *in vivo* immunosuppressive activity of the nanoformulation in a murine transplant model to determine its potential for clinic use.
